# Branchfall as a Demographic Filter for Epiphyte Communities: Lessons from Forest Floor-Based Sampling

**DOI:** 10.1371/journal.pone.0128019

**Published:** 2015-06-17

**Authors:** Juliano Sarmento Cabral, Gunnar Petter, Glenda Mendieta-Leiva, Katrin Wagner, Gerhard Zotz, Holger Kreft

**Affiliations:** 1 Biodiversity, Macroecology & Conservation Biogeography Group, Faculty of Forest Sciences and Forest Ecology, University of Göttingen, Göttingen, Germany; 2 Functional Ecology of Plants, Department of Biology and Environmental Sciences, University of Oldenburg, Oldenburg, Germany; 3 Smithsonian Tropical Research Institute, Panama City, Republic of Panama; DOE Pacific Northwest National Laboratory, UNITED STATES

## Abstract

Local variation in the abundance and richness of vascular epiphytes is often attributed to environmental characteristics such as substrate and microclimate. Less is known, however, about the impacts of tree and branch turnover on epiphyte communities. To address this issue, we surveyed branches and epiphytes found on the forest floor in 96 transects in two forests (Atlantic rainforest in Brazil and Caribbean rainforest in Panama). In the Brazilian forest, we additionally distinguished between edge and core study sites. We quantified branch abundance, epiphyte abundance, richness and proportion of adults to investigate the trends of these variables over branch diameter. Branches <2 cm in diameter comprised >90% of all branches on the forest floor. Abundance and richness of fallen epiphytes per transect were highest in the Brazilian core transects and lowest in the Panamanian transects. The majority of epiphytes on the floor (c. 65%) were found attached to branches. At all three study sites, branch abundance and branch diameter were negatively correlated, whereas epiphyte abundance and richness per branch, as well as the proportion of adults were positively correlated with branch diameter. The relationship between branch diameter and absolute epiphyte abundance or richness differed between study sites, which might be explained by differences in forest structure and dynamics. In the Panamanian forest, epiphytes had been previously inventoried, allowing an evaluation of our surveying method by comparing canopy and forest floor samplings. Individuals found on the forest floor corresponded to 13% of all individuals on branches <10 cm in diameter (including crowns), with abundance, richness and composition trends on forest floor reflecting canopy trends. We argue that forest floor surveys provide useful floristic and, most notably, demographic information particularly on epiphytes occurring on the thinnest branches, which are least accessible. Here, branchfall acts as an important demographic filter structuring epiphyte communities.

## Introduction

Vascular epiphytes are plants that grow on shrubs and trees, and thereby on a substrate distributed in three-dimensional space [1–3]. Microclimatic conditions change dramatically within this three-dimensional space, with generally drier and sunnier conditions towards the outer crowns of the trees (e.g. [4]). These conditions seem to cause higher drought-related mortality at early life stages compared to those in inner crowns and trunks [5]. Moreover, abiotic conditions vary within inner-crowns and between tree species [6–7]. These environmental gradients have been suggested to structure the distribution of epiphyte species [3–4, 7–10]. However, apart from the abiotic environmental conditions, the dynamics of the substrate itself should also influence epiphyte communities. This is because trees are constantly growing, producing new and losing older branches, meaning that the substrate persists only for a limited period [4, 11]. For example, trees commonly abscise branches lacking photosynthetically active leaves, which are more likely to be thin branches [12–13]. Moreover, thin branches stand less mechanical stress by epiphyte load, wind force, rainfall, or arboreal animals [9]. This might be particularly important in the outer crown of overstory trees, but the crown of understorey trees can also be disturbed by tree- and branchfall of large and emergent trees [14]. Such a highly dynamic system should have profound consequences for the population and community dynamics of vascular epiphytes. In fact, because thin branches fall more often than thick branches, epiphytes growing on these thin branches are particularly susceptible to substrate failure [15]. Correspondingly, only fast colonizing and maturing species are able to survive and reproduce on smaller branches [3, 16]. Branchfall may thus profoundly influence the distribution of epiphyte species within the canopy, contributing to niche partitioning in epiphyte communities.

Despite the apparent effects of diameter-dependent branchfall on community and population dynamics of epiphytes, pertinent studies are rare. Hietz [15] quantified mortality rates via branchfall by monitoring selected branches through tree climbing and repeated photography. However, this technique is costly, requires training and is time-consuming. These limitations are a general barrier to improve our understanding on epiphyte ecology, as the accessibility of tree canopy poses technical and logistic challenges. Among the techniques that are currently used to study epiphytes, the use of binoculars is the simplest [1, 17], whereas tree climbing [18–19], tree climbing and photographs (e.g. [15, 20]) and canopy cranes [21–22] demand considerable work and/or investment efforts. An alternative, inexpensive method to gather information on epiphyte demography is to sample the forest floor, particularly if combined with data on branchfall, a main cause of epiphyte mortality [15]. In fact, epiphytes on the forest floor may also provide information on the community structure and composition of epiphytes in tree crowns, but this data is also surprisingly scarce in the literature [23]. This lack of information is surprising, given that epiphytes on the forest floor could be further assessed for sustainable economic activities, such as gathering of fallen individuals for horticulture [23–24]. Hence, despite the conspicuous presence of epiphytes on the forest floor due to branchfall, this source of information has been largely neglected.

**Fig 1:**
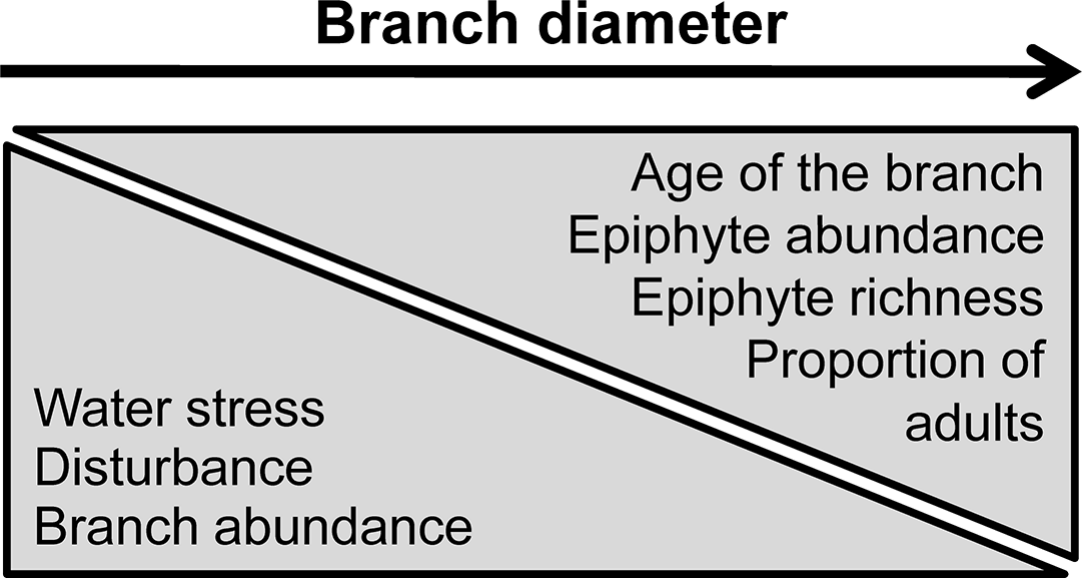
Summarizing scheme of the expected trends with increasing branch diameter. We expect a decrease in water stress, mechanical disturbance and branch abundance with an increasing branch diameter. These drivers plus the increase in branch age should lead to higher epiphyte colonization and survival and an increase in epiphyte abundance, richness and proportion of adults per branch.

To assess the usefulness of forest floor-based sampling to study vascular epiphytes, this study aimed to quantify branchfall and vascular epiphytes on the forest floor. The gathered information was used to assess the relationship of fallen epiphytes with branch diameter. For this purpose, we surveyed two Neotropical forests differing in epiphyte flora, elevation and climate (Atlantic submontane rainforest in northeastern Brazil and Caribbean lowland rainforest in Panama). In the former we distinguished edge and core forest habitats. For each study site, we addressed three hypotheses for epiphytes on the forest floor: 1) epiphyte abundance, 2) epiphyte richness and 3) proportion of adult epiphytes are positively correlated with branch diameter (Fig 1). In addition, we took advantage of data on precise three-dimensional positions of each individual epiphyte at the Panamanian site (Mendieta-Leiva, Wagner & Zotz, unpubl. data) to evaluate our sampling method and results by assessing how patterns on the forest floor relate to the canopy. For this purpose, we compared epiphyte abundance, richness and composition on forest floor and in the canopy. Overall, our results supported the hypotheses and demonstrated that branchfall-induced mortality has a non-negligible effect on the epiphyte community, particularly in the thin branches of tree crowns.

## Materials and Methods

### Study sites

We surveyed branches and vascular epiphytes on the forest floor at two Neotropical forests. The first forest was located within Usina Serra Grande, a large private sugar-cane landholding in the State of Alagoas, northeastern Brazil (8°58’50”S, 35° 54’30”W). It is part of the fragmented Brazilian Atlantic forest [25] which retains c. 90 km^2^ of forest of the Pernambuco Centre of Endemism [26], a unique biogeographic region within the Atlantic forest and a global biodiversity hotspot [27]. We studied a forest fragment of c. 50 ha surrounded by a uniform matrix of sugar-cane monoculture. The forest fragment was located at c. 550 m a.s.l. in a fairly flat hilltop terrain, lacking steep gorges or riverbeds. The area receives c. 2000 mm of rainfall per year with a 3-month dry season (<60 mm/month) from November to January and the wettest period is between April and August [28]. The forest can be classified as lower montane or submontane rainforest. The fragment harbors a rich epiphyte flora (11 species of bromeliads, 2 cacti, 31 orchids and 4 peperomias; ferns and aroids have not been studied—[29–30]). The fragment has a relatively old and stable edge (> 80 years), whose effects can be detected up to 100 m from the forest borders [28]. We sampled both forest core and edge habitat. These two habitats are referred to as Brazilian core and Brazilian edge study sites.

The second forest and our third study site was the San Lorenzo Canopy Crane plot located near the Atlantic coast of the Republic of Panama (9°16'50”N, 79°58’30”W, [31]). The site is part of one of the largest undisturbed forest landscapes in Panama. It is at c. 130 m a.s.l. and receives c. 3500 mm of rainfall per year, with a three- to four-month dry season (<60 mm/month) between January and April. The epiphyte flora has already been described in detail (>90 species of holoepiphytes; [22]). At the crane site, only core forest conditions could be sampled, due to land mines from the US-American period of military training in the area outside the field station. The crane site is located in a narrow valley with partially steep slopes over 45° of inclination, with a centrally-located and seasonally dry creek. The proximity to the Caribbean coast also exposes the area to frequent heavy storms and associated disturbances. The total area covered by the crane plot is c. 0.9 ha (more details of the study site in [3, 22]).

Field work in the Brazilian study sites was supported by the Federal University of Pernambuco, which has a research agreement with the landholding that owns the forest fragments. The permit for the Panamanian study site was obtained from the Panamanian Environmental Agency (ANAM) via Smithsonian Tropical Research Institute (STRI). Field work was done for four weeks in each country: July 2012 (Brazil) and in September/October 2012 (Panama). The surveyed period coincided with the second half of the rainy season in each forest, and thus we were able to sample branches freshly broken due to heavy storms. This was important, as fallen epiphytes may die within a few months after branchfall, but can live up to a year [32].

### Branchfall

Surveys of branches on the forest floor were conducted within randomly placed 5 x 0.5 m transects (60 in Brazil, 36 in Panama). In Brazil, 30 transects were placed at least 200 m from the forest edge (from now on called 'Brazilian core transects'); 30 transects were placed within 60 m of the forest edge ('Brazilian edge transects'). In each of the 96 transects, we screened the forest floor for branches. We counted only branches with > 50% of their length within transects and that did not crumble due to advanced decomposition when handled. Branches were divided into four diameter classes based on the thickest internode (0.5–2, 2–4, 4–6, >6 cm). Number and size of side branches were ignored. Branches with < 0.5 cm diameter were surveyed in 1 x 0.5 m subplots nested and centrally located in each transect, and their number was extrapolated from the subplot to the 5 x 0.5 m transect.

We follow the terminology presented by [33] for the terms 'canopy' (aboveground parts, including tree crowns and trunks) and 'crown' (branches, excluding the trunk). We were not able to identify the origin of the branches found on the forest floor. Hence, although most thin branches can be assumed to have their origin in the outer crowns of overstorey trees, they could also be from understorey trees and shrubs as well as from inner crowns. We did not count thin branches attached to thick ones, but it is reasonable to assume that at least some thin branches detach during descent or upon impact on the forest floor. This detachment of thin branches should thus increase their abundance on the forest floor.

### Epiphytes

We extended the 96 branch transects longitudinally to 5 x 10 m and surveyed vascular holoepiphytes on the forest floor. For each individual, we recorded species identity, life stage (juvenile or adult), and diameter of host branch if present. Classification as adult was based on remains of inflorescence and/or size comparable to reproductive conspecifics. We sampled all epiphyte taxa in Panama, but excluded ferns and aroids at Brazilian transects due to difficulties with species identification. The full sampling in Panama allowed the comparison between forest floor and canopy besides addressing branchfall effects on epiphyte community, whereas Brazilian transects were mainly used for addressing branchfall effects. Excluding ferns and aroids from the Panamanian transects did not change the relationships of epiphyte community variables (abundance, richness and proportion of adults) with branch diameter (results not shown). This suggests that the absence of ferns and aroids in the Brazilian transects should not affect the analysis of branchfall effects. A list of observed vascular holoepiphyte species is provided in S1 Table.

Epiphytes species in the canopy at the Brazilian sites were surveyed using a combination of ground-based observation with binoculars and tree climbing (S2 Table). In Panama, the epiphytes occurring in the canopy had recently been surveyed in a comprehensive census from 2010–2012, in which the precise identity and the host branch diameter of every individual epiphyte was recorded (Mendieta-Leiva, Wagner & Zotz, unpubl. data; S2 Table).

As additional structural characteristic of each transect, we determined diameter at breast height (DBH), mean height at first branching (first ramification of the stem) and total tree height for all trees with DBH > 5 cm.

### Analyses

First, we quantified mean values of key physiognomic variables of the forest for each transect (number of trees, tree DBH, height at first branching, tree height), as well as of branch abundance, epiphyte abundance and epiphyte richness on the forest floor. We additionally quantified mean values of abundance and richness for epiphytes attached to branches, detached from branches and adult individuals. For epiphytes attached to branches, we further quantified mean values of epiphyte abundance and richness per branch in each transect. We accounted for differences in area between epiphyte and branch transects (50 and 2.5 m^2^, respectively) by multiplying the number of branches found in the branch transects by 20. For all variables, we compared the three study sites (Brazilian core, Brazilian edge, Panamanian transects) with simultaneous max-*t* tests using Tukey contrasts that are robust under non-normality, heteroscedasticity and variable sample size [34]. For adequate comparisons, ferns and aroids were excluded from Panamanian transects.

To investigate the effect of sampling effort on species numbers, we generated species accumulation curves per study site by randomizing 100 times the increase in species richness caused by subsequently adding one transect to the sample. Species accumulation curves tending to an asymptotic value (near the actual number of species) reveal appropriate sampling effort.

Using the branches on the forest floor, we addressed whether the assumption that branch abundance on the forest floor is negatively correlated with branch diameter was true. Due to possible non-linear relationships with branch diameter (e.g. [15]), we used generalized additive mixed-effects models (GAMMs) with the absolute number of branches per transect as response, branch diameter class as fixed effect and transect as random effect [35]. Transect was used as random effect because branch abundances varied between transects, probably reflecting variation in age, structure and abscission patterns of local tree species. We applied negative binomial GAMMs (with log link function) to account for possible overdispersion in count data [35–37]. Thereafter, we addressed the hypotheses that 1) epiphyte abundance, 2) epiphyte richness and 3) proportion of adult epiphytes are positively correlated with branch diameter (Fig 1). Similarly to branch abundance, we performed GAMMs with the same fixed and random effects. As response variables, we first used absolute number of individuals per transect (referred to as absolute epiphyte abundance) and absolute number of species per transect (i.e. absolute epiphyte richness). In both cases, we applied negative binomial GAMMs. Second, to adequately test the hypotheses given potential differences in branch abundance, we performed GAMMS controlling for branch abundance per diameter class in epiphyte abundance and richness. To this end, we standardized both epiphyte abundance and richness by dividing them by branch abundance (from now on referred to as abundance per branch and richness per branch, respectively). These two variables were used as response in gamma family GAMMs with log link function [35]. Finally, the proportion of adults was used as response variable for binomial GAMMs [35].

We further assessed whether epiphyte abundance and richness observed on the forest floor reflect the trends observed in the canopy (trunk and crowns). To address this question, we analyzed the epiphyte abundance and richness in branch diameter classes in the canopy directly above the Panamanian transects. For this purpose, we used the vascular epiphyte inventory of the crane plot ([22], Mendieta-Leiva, Wagner & Zotz, unpubl. data). From our 36 Panamanian transects, 29 had their canopy epiphytes inventoried. Similarly to the analyses of the forest floor, we applied negative binomial GAMMs (with log link function) with epiphyte abundance and richness as response variables, branch diameter class as fixed effect and transect as random effect [35]. Additionally, we used Spearman correlations to test whether abundance and richness on the forest floor were correlated with their canopy counterparts. For these correlations, two analyses were performed: i) per transect (all epiphytes found on the forest floor and inventoried in the canopy) and ii) per transect and per branch diameter class (only epiphytes found on the forest floor attached to branches and canopy epiphytes on substrate with the same diameter distribution as on the forest floor). Thereafter, we assessed the proportion of the epiphytes over branch diameter found on the forest floor in relation to the entire transect (floor and canopy). For this analysis, we applied binomial GAMMs with the proportion of individuals and species on the forest floor as response variables, branch diameter class as fixed effect and transect as random effect [35].

The species composition found on the forest floor was compared with that from the inventoried canopy above Panamanian transects. We compared 1) all epiphytes and 2) epiphytes found only on branches < 10 cm in diameter. Initially, we built a species per transect matrix with abundances separately for ground and for canopy individuals. To avoid bias due to low richness on the forest floor per transect, but still retain a reasonable number of transects, we included only transects with at least two species on the ground (n = 18 considering all epiphytes, n = 17 considering only epiphytes on branches < 10 cm in diameter). We performed a non-metric multidimensional scaling (NMDS) and plotted the resulting ordination showing separate convex hulls for ground and canopy. For this analysis, we estimated a dissimilarity matrix (Bray-Curtis index) between transects. Thereafter, we used this dissimilarity matrix to perform an analysis of similarities (ANOSIM) between ground and canopy. We then assessed which species were responsible for significant differences between ground and canopy by performing a Dufrene-Legendre indicator species analysis [38]. Finally, we tested whether paired ground and canopy transects were more similar than expected by chance. For this analysis, we estimated the Bray-Curtis dissimilarity index for each transect pair and for random pairs (n = 18 pairs considering all epiphytes, n = 17 considering only epiphytes on branches < 10 cm in diameter). The dissimilarity of each random pair was an average of the dissimilarity between each ground sample and n random canopy samples other than its actual canopy sample. We then compared the mean dissimilarity between actual vs. random pairs with simultaneous max-*t* tests using Tukey contrasts [34].

All analyses were done in R (version 3.0.1). GAMMs were implemented using the R library ‘mgcv’ version 1.7–24 [39]. Ordination, dissimilarity matrices and analyses of similarities were implemented using the R library 'vegan', whereas Dufrene-Legendre indicator species analyses used the library 'labdsv'. We provide raw data and R codes as: S1 File for R codes, S1 Dataset for tree data, S2 Dataset for branch data, S3 Dataset for epiphyte data on the forest floor and S4 Dataset for epiphyte canopy data.

## Results

**Fig 2:**
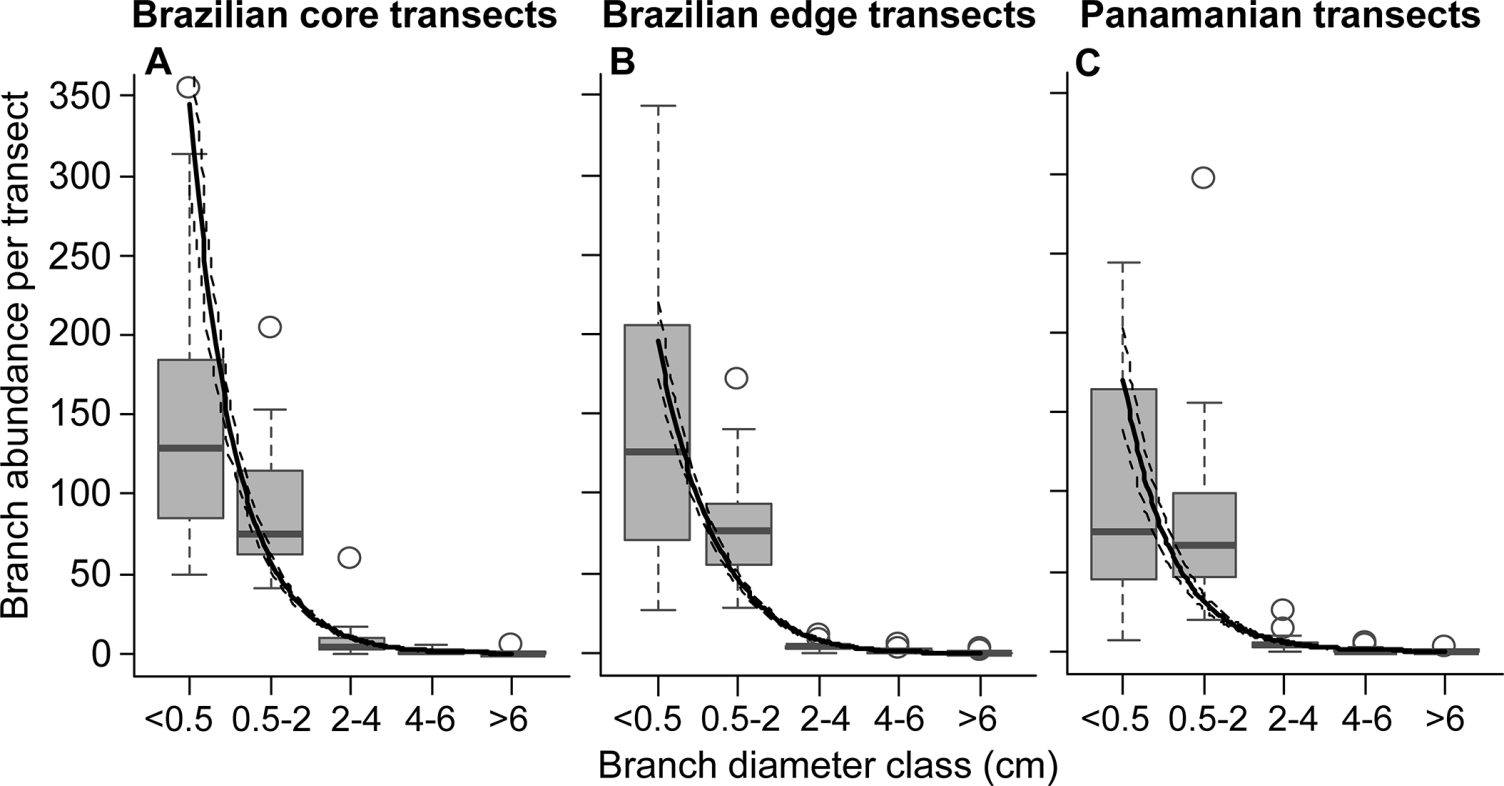
Branch abundances as a function of branch diameter. A) Brazilian core transects (n = 30). B) Brazilian edge transects (n = 30). C) Panamanian transects (n = 36). Box-plots show the median as central line, the first and third quantiles as the bottom and top box limits, 1.5 interquantile range as whiskers, and outliers as circles. Solid lines show fits from GAMMs with 95% CI indicated by dashed lines.

In total, we counted >24,000 branches at the two sites. Brazilian core transects had 325 ± 284 (mean ± SD, n = 30) branches per transect, Brazilian edge transects had 224 ± 102 (n = 30) branches per transect and the 36 Panamanian transects 220 ± 169 branches per transect (equivalent to an average of 130, 90 and 88 branches per m^2^, respectively). Although Brazilian core transects had, on average, the highest number of branches, branch abundance did not differ significantly between study sites (Table 1; see S3 Table for an extended version of Table 1). At all three study sites, the abundance of the thinnest branches was significantly higher than those of thicker ones, with > 90% of all branches belonging to the first two diameter classes (<0.5 cm and 0.5–2 cm, Fig 2; Table 1).

**Table 1:** Comparisons between study sites. Several measures characterizing forest structure, branch abundance, epiphyte abundance and epiphyte richness.

		Brazilian core transects (*n* = 30)	Brazilian edge transects (*n* = 30)	Panamanian transects (*n* = 36)	
Variable				No ferns and aroids	All species
**Trees**	**Mean number per transect**	5.1 ± 2.1 ab	6.4 ± 3.2 a	‒	4.8 ± 2.1 b
	**Mean DBH (m)**	0.2 ± 0.1 a	0.18 ± 0.06 b	‒	0.16 ± 0.06 b†
	**Mean height at first branching (m)**	8.2 ± 2.0 a	4.7 ± 2.0 b	‒	8.0 ± 2.0 a†
	**Mean height (m)**	15.4 ± 3.4 a	10.9 ± 2.7 b	‒	13.2 ± 2.6 c†
**Branch abundance**	**Total**	9759	6721	‒	7939
	**Mean per transect**	325 ± 284 a	224 ± 102 a	‒	220 ± 169 a
	**Mean per transect (< 0.5 cm diameter)**	215 ± 237 a	142 ± 85 a	‒	135 ± 151 a
	**Mean per transect (< 2 cm diameter)**	316 ± 283 a	219 ± 101 a	‒	214 ± 168 a
**Epiphyte abundance**	**Total**	546	349	164	232
	**Total adults**	211 (39%)	153 (44%)	86 (52%)	101 (44%)
	**Total attached to branches**	367 (67%)	260 (74%)	112 (68%)	164 (71%)
	**Mean per transect**	18.2 ± 20.5 a	11.6 ± 17.8 ab	4.6 ± 7.1 b	6.4 ± 9.8
	**Mean attached to branches per transect**	12.2 ± 17.0 a	8.7 ± 16.8 ab	3.1 ± 5.1 b	4.6 ± 6.4
	**Mean attached to branches per transect per branch**	2.8 10^–3^ ± 3.5 10^–3^ a	2.3 10^–3^ ± 4.5 10^–3^ a	1.1 10^–3^ ± 2.0 10^–3^ a	1.8 10^–3^ ± 3.1 10^–3^
**Epiphyte richness**	**Total**	23	16	27	39
	**Total adults**	21 (91%)	14 (88%)	17 (63%)	24 (62%)
	**Total attached to branches**	17 (74%)	13 (81%)	18 (67%)	29 (74%)
	**Mean per transect**	5.1 ± 3.2 a	2.3 ± 1.9 b	1.9 ± 2.3 b	2.8 ± 3.6
	**Mean attached to branches per transect**	3.3 ± 2.5 a	1.8 ± 1.8 b	1.4 ± 1.6 b	2.2 ± 2.3
	**Mean attached to branches per transect per branch**	7.7 10^–4^ ± 7.1 10^–4^ a	5.2 10^–4^ ± 5.5 10^–4^ a	5.3 10^–4^ ± 7.5 10^–4^ a	7.3 10^–4^ ± 7.9 10^–4^

† indicates n = 35 (excluding one transect without trees)

Total numbers and means ± SD per study site are provided. Percentages of adults and epiphytes attached to branches to the study site totals are given in parentheses. Means were compared with simultaneous max-*t* tests using Tukey contrasts that are robust under non-normality, heteroscedasticity and variable sample size [34]. Significantly different means are indicated by different letters representing pairwise differences. Note that for epiphytes, only mean values for Panamanian transects without ferns and aroids were used in the comparisons with Brazilian study sites.

**Fig 3:**
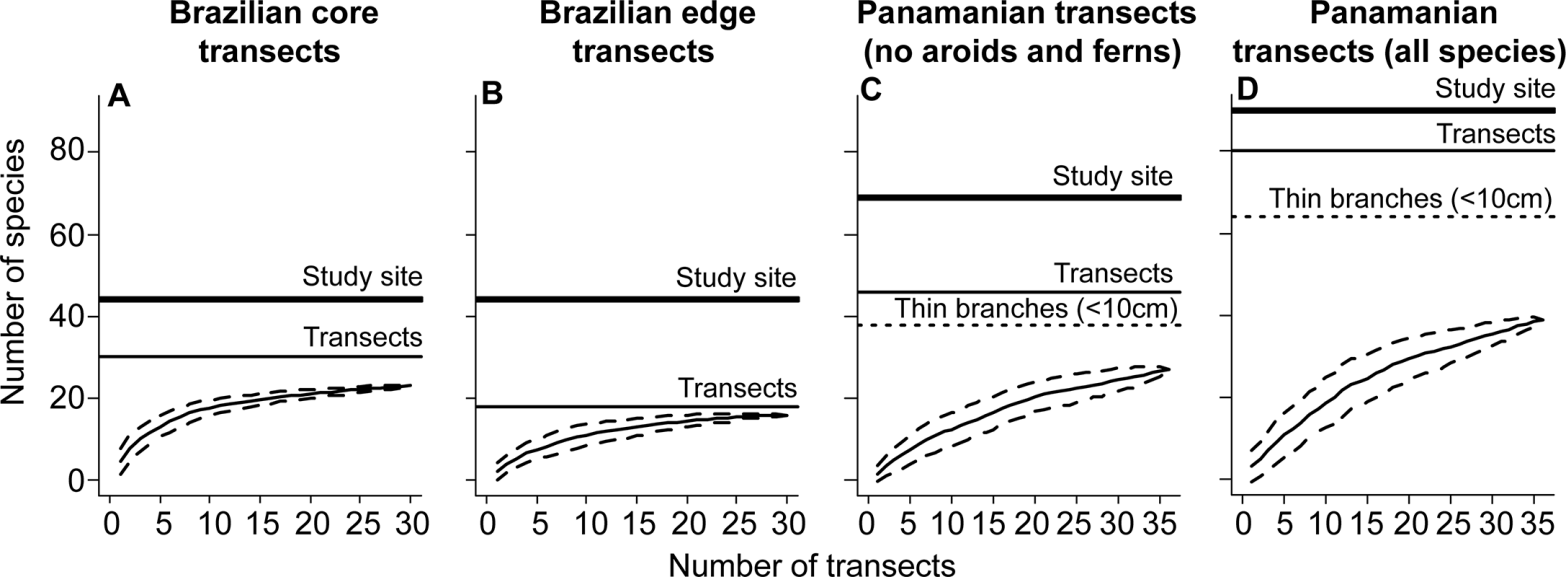
Species accumulation curves based on forest floor-based sampling of epiphytes. A) Brazilian core transects (n = 30). B) Brazilian edge transects (n = 30). C) Panamanian transects, excluding ferns and aroids (n = 36). D) Panamanian transects, all species (n = 36). Solid curves give the mean number of species based on 100 randomized samplings, dashed curves the 95% CI. Horizontal lines indicate the number of species present in the canopy of the transects (thin lines), in the study site (thick lines, same estimate for both Brazilian study sites) and on substrate < 10 cm in diameter (dot line, in c and d). See S1 Table for the list of species found in the transects and S2 Table for species lists found in the study sites. Note that the Brazilian study sites showed curves leveling off, whereas the Panamanian site revealed a slightly steeper curve in agreement with the higher number of species.

We found a total of 546 individuals of 23 epiphyte species in Brazilian core, 349 individuals of 16 species in Brazilian edge, and 232 individuals of 39 species in Panamanian transects (Table 1; see S1 Table for species lists). Overall, the transects captured a considerable proportion of the epiphyte species found in the forests of the respective study sites (36–52%, Fig 3). When considering only the species in the transects' canopy, a larger proportion of epiphytes species was found on the forest floor (49–89%, Fig 3). Excluding ferns and aroids of the Panamanian transects for comparisons between study sites, absolute epiphyte abundance was significantly higher in Brazilian core transects (c. 18 individuals per transect) compared to Brazilian edge (12 individuals per transect) and Panamanian (5 individuals per transect) transects (Table 1; equivalent to c. 0.36, 0.23 and 0.11 individuals per m^2^, respectively). Similarly, the average absolute species richness per transect was significantly higher in Brazilian core transects (c. 5 species per transect) than in Brazilian edge (2.3 species per transect) and Panamanian (c. 2 species per transect) transects (Table 1; equivalent to c. 0.1, 0.05 and 0.04 species per m^2^, respectively). However, differences in epiphyte abundance and richness, when standardized per branch, were not significant (Table 1).

Most individuals fell attached to branches (>65%), particularly in the Brazilian edge transects (Table 1). Epiphytes detached from branches were often attached to bark pieces, canopy soil or moss mats. Between 39% and 52% of the individuals on the forest floor were adults (Table 1). Adults were found for most of the species, particularly at the Brazilian core transects (91% of species; Table 1). Remarkably, the proportion of adults among those individuals detached from branches was much higher (56% in Brazilian core, 64% in Brazilian edge, and 72% in Panamanian transects) than among those attached to branches (30% in Brazilian core, 37% in Brazilian edge, and 36% in Panamanian transects; see S3 Table for total numbers).

**Fig 4:**
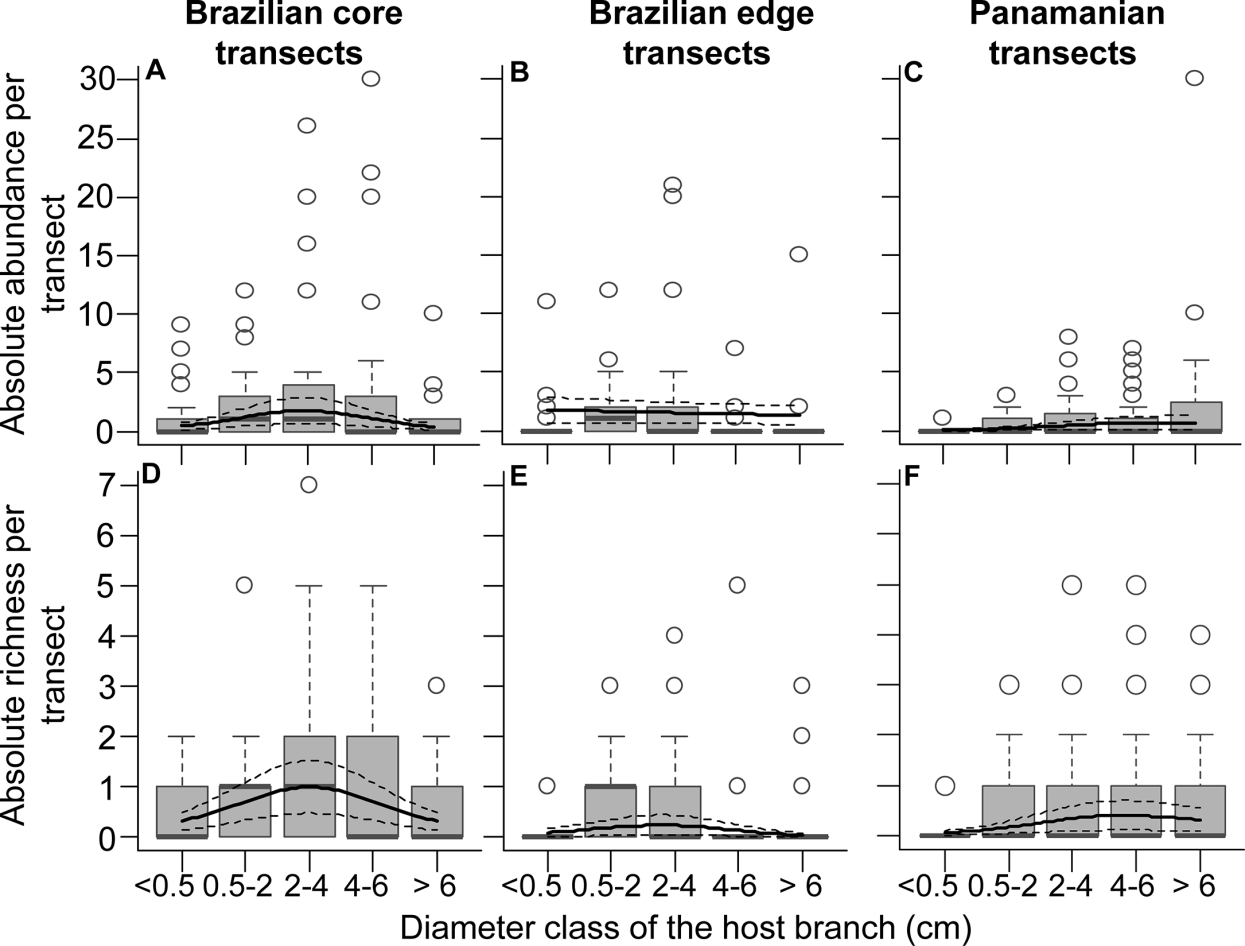
Absolute epiphyte abundance (A-C) and richness (D-F) per transect as a function of branch diameter. Trends are shown for Brazilian core (A,D, n = 30), Brazilian edge (B,E, n = 30), and Panamanian (C,F, n = 36) transects. Box-plots show the median as central line, 1.5 interquantile range as whiskers, and outliers as circles. Solid lines give the values predicted by the estimated GAMMs, dashed lines show 95% CI. Note that the number of epiphytes was generally hump-shaped along diameter classes in Brazilian transects, whereas it was positive in Panamanian transects.

**Fig 5:**
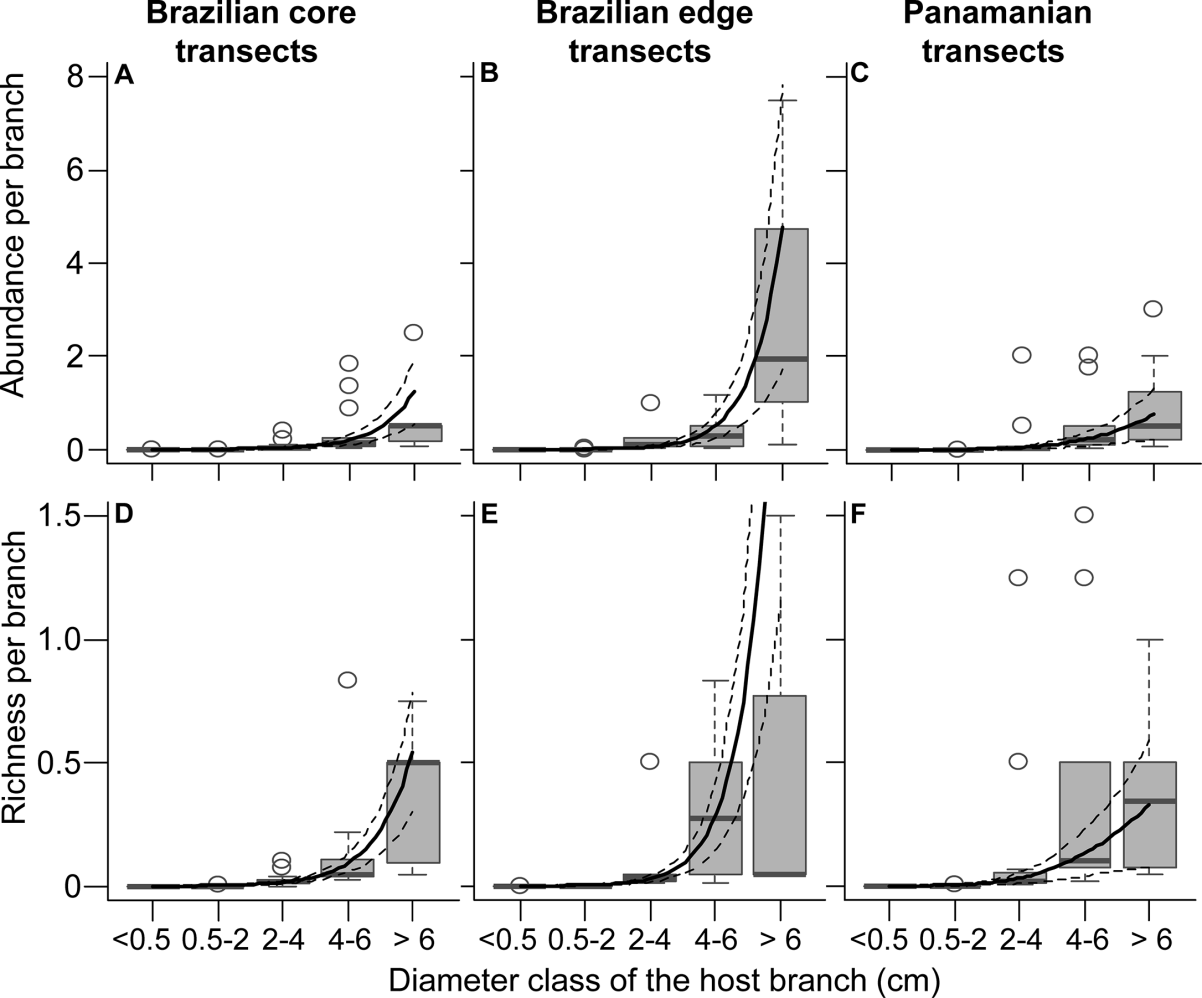
Epiphyte abundance (A-C) and richness (D-F) per branch as a function of branch diameter. Trends are shown for Brazilian core (A,D, n = 26), Brazilian edge (B,E, n = 21), and Panamanian (C,F, n = 25) transects. Box-plots show the median as central line, 1.5 interquantile range as whiskers, and outliers as circles. Solid lines give the values predicted by the estimated GAMMs. Dashed lines show 95% CI.

**Fig 6:**
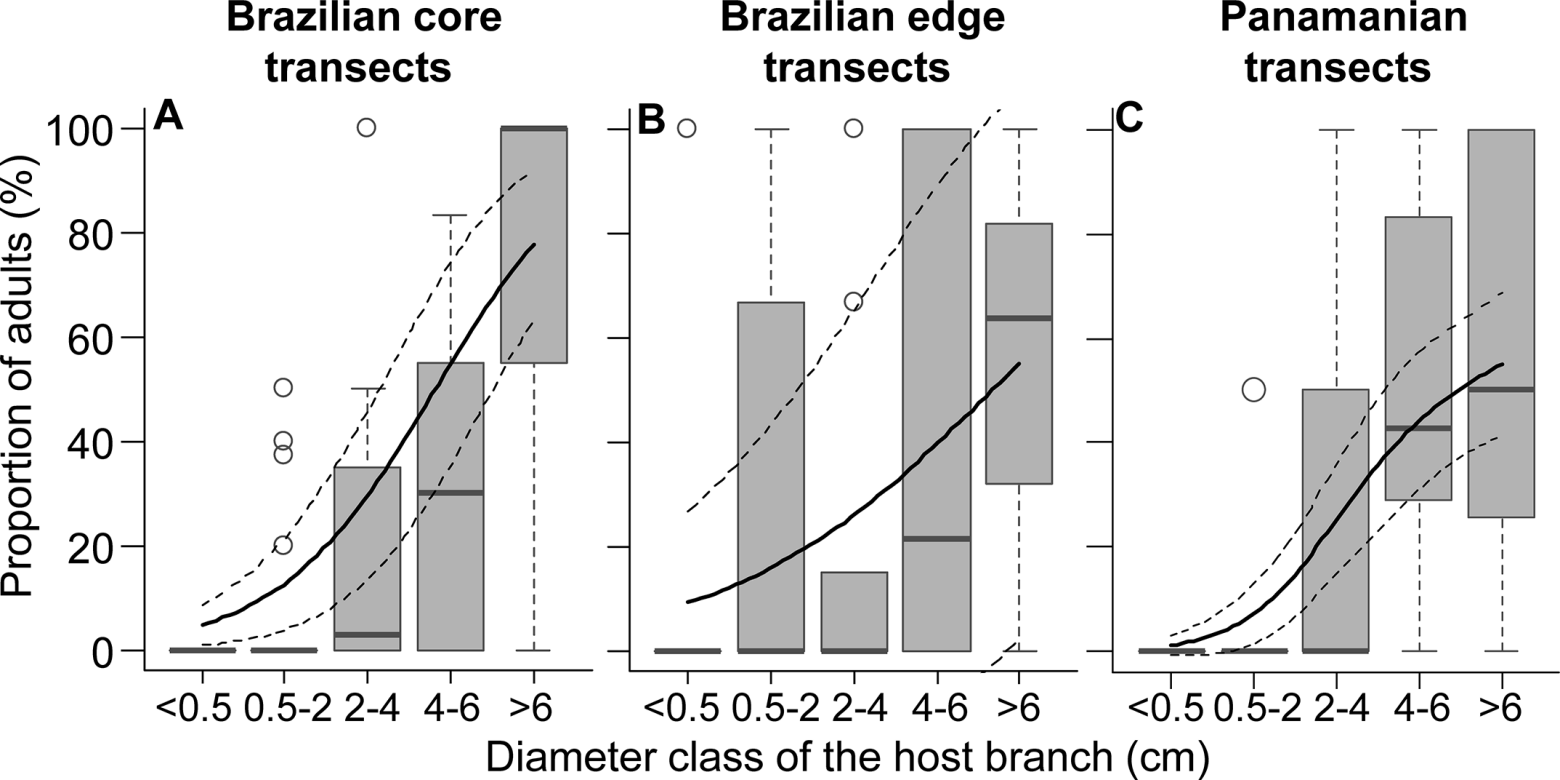
Proportion of adults as a function of branch diameter. A) Brazilian core transects (n = 30). B) Brazilian edge transects (n = 30). C) Panamanian transects (n = 36). Box-plots show the median as central line, 1.5 interquantile range as whiskers, and outliers as circles. Solid lines give the values predicted by the estimated GAMMs. Dashed lines show 95% CI.

The relationship between absolute epiphyte abundance or richness and branch diameter differed between study sites (Fig 4; see S4 Table for summary statistics). Absolute epiphyte abundance showed a hump-shaped relationship with increasing branch diameter in Brazilian core transects (Fig 4A), with no clear relationship in Brazilian edge transects (Fig 4B) and a positive relationship in Panamanian transects (Fig 4C). Absolute species richness showed a hump-shaped relationship with increasing branch diameter at both Brazilian study sites (Fig 4D and 4E) and a positive relationship in the Panamanian transects (Fig 4F). In contrast to these trends, the abundance (Fig 5A–5C) and richness (Fig 5D–5F) per branch showed a positive relationship with branch diameter at all three study sites (Fig 5, S4 Table). There was a positive relationship between proportion of adults and branch diameter at all three study sites (Fig 6, S4 Table).

**Fig 7:**
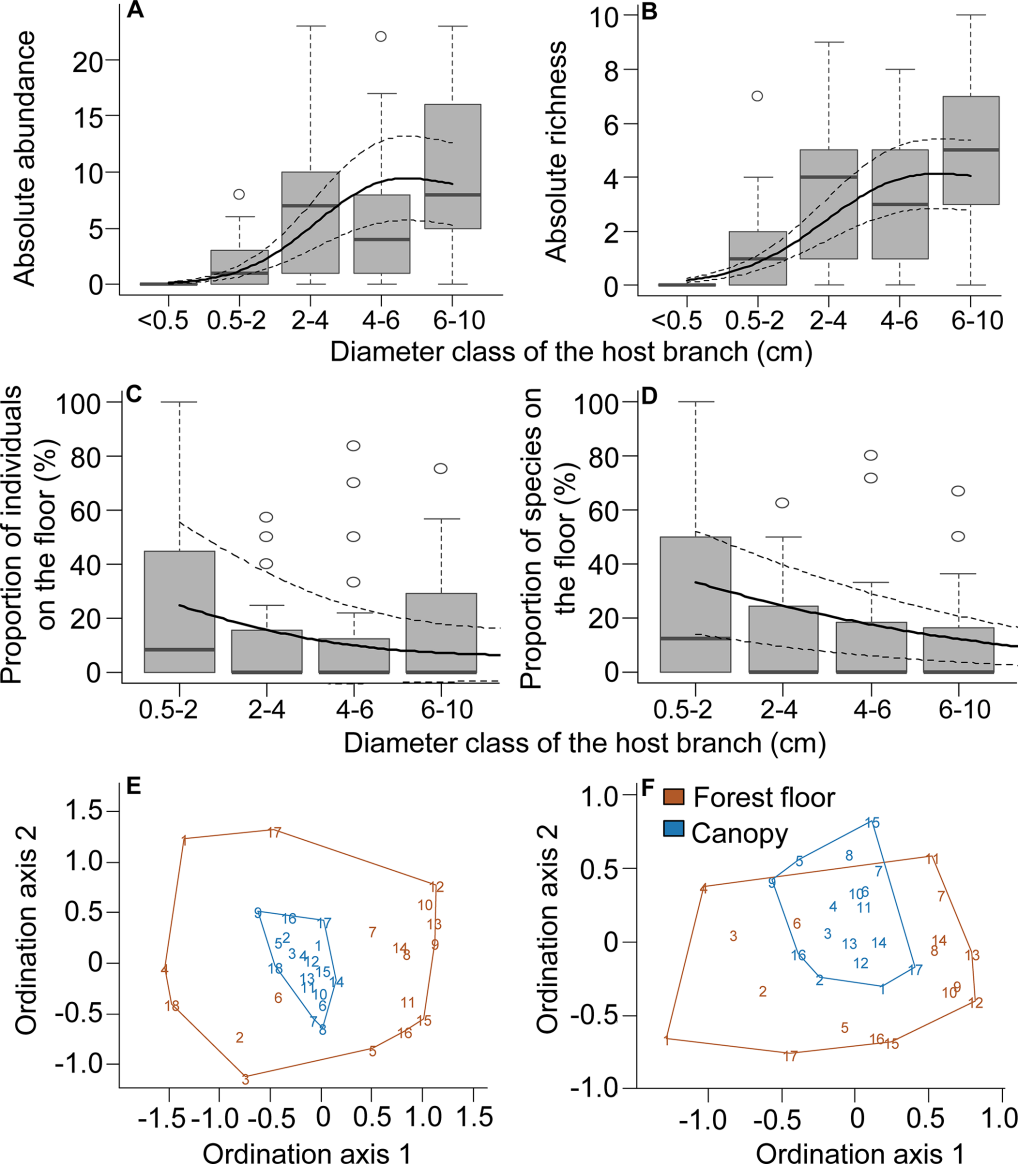
Epiphytes in the canopy and their relationship with forest floor trends. (A) epiphyte abundance and (B) species richness in the canopy directly above the Panamanian transects (n = 29) as a function of branch diameter. Proportion of individuals (C) and species (D) found on the forest floor compared to the transects' total abundance (forest floor and canopy). (E-F) Non-metric multidimensional scaling of transects based on species composition and abundance considering (E) all individuals found on the forest floor and canopy (n = 18 forest floor and canopy pairs) and (F) only individuals on substrate < 10 cm in diameter (n = 17). Forest floor and canopy pairs are indicated by the same numbers in E-F (legend in F). See S2 File for the number of individuals and species censed within the whole crane plot (c. 0.9 ha). Solid lines give the values predicted by the estimated GAMMs, whereas dashed lines show 95% CI in A-D. Lines connecting numbers indicate convex hulls in D-F. We excluded the thinnest branch diameter class in C and D due to overall low abundances in the canopy (see A-B). Box-plots show the median as central line, 1.5 interquantile range as whiskers, and outliers as circles.

The inventoried canopy above our Panamanian transects had 4386 epiphyte individuals (corresponding to 151 individuals per transect or ~3 individuals per m^2^) of 80 species. Considering only substrates with diameters comparable to those found on the forest floor (<10 cm), the inventoried canopy above our transects hosted 866 epiphyte individuals (30 individuals per transect, 0.6 individuals per m^2^) belonging to 64 species (20% of all individuals and 80% of all species). Epiphyte abundance (*P* <0.001, df_eff_ = 1.98) and richness (*P* <0.001, df_eff_ = 1.97) above the Panamanian transects were positively related to increasing branch diameter (Fig 7A and 7B). There was no significant correlation between the number of individuals and species on the forest floor and in the canopy. Across Panamanian transects, epiphytes on the forest floor (both attached to or detached from branches) corresponded to c. 4% of total number of individuals and to 48% of the species found in the entire transect (forest floor + canopy). When comparing only those individuals attached to branches < 10 cm in diameter, this proportion was of 13% for individuals and 40% for species, gradually decreasing with branch diameter for individuals (*P* <0.05, df_eff_ = 1.53) and species (*P* <0.001, df_eff_ = 1.00; Fig 7C and 7D). Species composition differed significantly between ground and canopy for all epiphytes (*P* = 0.001, ANOSIM statistic R = 0.37) and for epiphytes on substrate < 10 cm in diameter (*P* = 0.001, ANOSIM statistic R = 0.29). Species composition on the ground was more variable than in the canopy (Fig 7E and 7F), particularly considering all epiphytes (Fig 7E). Most indicator species of these compositional differences were aroids and ferns that are found only or mostly in the canopy, including trunks (S5 Table). Mean species similarity between actual ground and canopy transect pairs was not significantly different from the mean species similarity between random ground and canopy pairs.

## Discussion

### Surveying epiphytes on the forest floor

A considerable proportion of the species above the transects was also found on the forest floor (Fig 3, Fig 7C and 7D). Single transects entailed random subsets of the epiphyte community in the canopy (Fig 7E and 7F). Although this may be the main limitation of this method, at least some species typically restricted to stable substrates (i.e. tree trunks and inner crowns, [3–4]) were found on the forest floor. In fact, most of the individuals found detached from branches were attached to substrate parts (e.g. bark pieces or canopy soil) and thus may have fallen from trunks and inner crowns. Most indicator species for the Panamanian canopy transects preferentially occur, however, on trunks and inner-crowns (e.g. *Trichomanes* spp., *Campyloneurum* spp., *Anthurium* spp., *Dichaea panamensis*; full list in S5 Table). Hence, to increase the effectiveness and completeness, forest floor-based surveys could target transects with fallen trees or near old trees, as old trees have been indicated to host a higher number of species and should always be included in epiphyte surveys [40–41].

Sampling the forest floor might be particularly useful for investigating epiphytes occurring on branches < 10 cm in diameter. This is illustrated by the fact that there were considerably fewer canopy indicator species when limiting the comparison between canopy and forest floor to branches < 10 cm in diameter than when considering the entire canopy (S5 Table). This is also supported by the lower species number (Fig 3C and 3D) and variation in composition (Fig 7E and 7F) compared to similar analyses considering epiphytes of the entire canopy. Furthermore, an unexpectedly high proportion of epiphytes on branches < 10 cm in diameter were found on the forest floor (13% of all individuals belonging to 40% of all species, see also Fig 7C and 7D for averages over each diameter class). This is important because these thin branches, often located in the outer crowns, are the most difficult canopy habitats to access despite hosting a sizable portion of individuals and species (20% and 80%, respectively, for Panamanian transects).

In addition to floristic information, the forest floor was an important source of information on epiphyte demography and community structure. The fact that the patterns of epiphyte abundance and richness over branch diameter found on the forest floor mirrored the community structure of the canopy (compare Figs 4 and 5, 7) indicates that the community structure of the canopy can be surveyed on the forest floor. Hence, demographical inferences can be attempted with a survey method that is faster, cheaper and safer than commonly applied techniques, such as tree climbing and research cranes (see also [23]). This is valuable information, considering that even if floristic data from the forest floor might not be as complete as from tree climbing, the forest floor offers much needed demographic data. Furthermore, besides surveys focusing on economic value of fallen epiphytes [23–24], further studies incorporating forest floor information can focus on combining demography and community structure with substrate characteristics.

### Epiphyte fall and branch diameter

We found a high density of epiphytes on the forest floor (1100–3600 individuals per hectare). The fact that most epiphytes on the forest floor were found attached to branches emphasises the importance of branchfall as a cause of epiphyte mortality. Although we have not directly measured mortality rates via falling with or from branches [42], indirect estimates are possible if epiphytes in the canopy have been inventoried, as in our Panamanian study site. In this case, the mortality rate caused by falling with or from branches would be at least 4% per year (percentage of individuals found on the forest floor), assuming that the majority of epiphytes on the forest floor dies within less than one year [32]. Our estimate is lower than the annual mortality rate due to epiphyte fall reported for a humid montane forest via monitoring selected branches with photographs (16%, [15]). However, epiphyte abundances in the canopy of the Panamanian study site were generally low compared to montane cloud forests [10, 42], which may contribute to branchfall [9]. Nevertheless, our estimate of 13% mortality rate when considering only branches < 10 cm in diameter is more comparable to the reported 16% [15], particularly because Hietz [15] did not monitor trunks and observed epiphyte fall only with or from branches < 12 cm.

When considering only epiphytes falling with branches, absolute epiphyte abundance and richness revealed site-specific types of relationships with branch diameter (Fig 4). As such differences disappeared after accounting for branch abundance (Fig 5), they likely reflect differences in local branch dynamics. The resulting epiphyte abundance and richness per branch supported the hypotheses of higher abundance and richness on thick branches (compare Figs 1 and 5). The main explanation for higher epiphyte abundance and richness on branches of larger diameter classes is lower epiphyte mortality via branchfall (also found by [15]) and more time for colonization. In fact, branchfall was identified as the main cause of epiphyte fall (Table 1) and the assumption that thin branches are more abundant on the forest floor than thicker ones was confirmed (Fig 2; [9, 13, 43–44]). Furthermore, thicker branches support a micro-environment that is more suitable for the epiphytic lifestyle, with lower mortality at the seedling stage due to lower exposure to wind, lower water stress [5] and more suitable substrate properties, such as higher moss cover, humus volume and humus layer thickness [4, 8]. As a consequence, epiphyte richness, cover and biomass are usually higher on the thicker branches of the inner crowns [3, 10, 15]. Accordingly, higher epiphyte abundance and richness on thicker rather than thin branches were also observed in the canopy at the Panamanian site (compare Figs 4 and 5 to Fig 7A and 7B; see also S2 File for epiphytes inventoried within the entire crane plot). However, quantifying branch abundance, as done for the forest floor but not for the canopy, seems essential to account for the effects of site-specific branch dynamics on the gradients of epiphyte abundance and richness over branch (compare Figs 4 and 5).

Our hypothesis of a positive correlation between the proportion of adults and branch diameter was also supported (compare Figs 1 and 6), indicating a strong role of branchfall on the spatial structuring of epiphyte populations. Consequently, most adults in the outer crowns are twig epiphytes with fast life-cycles [16, 45]. In fact, most adults in the two thinnest diameter classes were from small species classifying as twig epiphytes: *Campylocentrum crassyrhyzum*, *Rodriguezia bahiensis* and *Notylia lyrata* in Brazilian transects as well as *Campylocentrum micranthum* in Panamanian transects.

### Study sites

We found small site-related differences in total species richness (Table 1) and in species-accumulation curves (Fig 3). The lower total species richness of edge transects was likely associated with the fact that most species absent in the edge have long life cycles, requiring at least 10 or more years to reproduce (e.g. *Maxillaria ochroleuca*, *Prosthechea fragrans*—first author's observations based on pseudobulb and inflorescence skeletons), or are probably less tolerant to water-stress (e.g. *Anathallis sclerophylla*, *Acianthera pernambucensis*, which were observed only on moss-rich shaded substrate). Consequently, the lack of large, stable, old trees and dominance of fast-growing pioneer trees at the same studied edge compared to core site [46–47] may reduce the establishment and survival of late-maturing and moisture-demanding epiphytes due to greater substrate dynamics and drier microclimate [48]. Such lower colonization would explain why our forest floor-based sampling detected almost all species present in the canopy of edge transects but not in that of core transects (Fig 3). However, because we only have one edge and core pair, we cannot statistically compare edge vs. core due to pseudoreplication, and thus further studies incorporating more pairs are necessary to investigate to what extent edge conditions affect epiphyte community composition.

The total observed epiphyte richness at the Panamanian study site in turn was slightly higher than at the Brazilian core (Table 1, Fig 3). However, at the transect scale, Panamanian transects had fewer species than the Brazilian transects (Table 1). High total species richness but low richness at the transect scale indicates a high spatial turnover of epiphyte species in Panama. This high turnover might be associated with increasing turnover of aboveground biomass with decreasing elevation [49]. Considering single trees or branches as habitat patches, local communities are colonized by species occurring in adjacent patches (higher recruitment near source areas—[50]). Hence, an increase in turnover of such patches might preclude the accumulation of species. This idea is supported by the high number of fallen trees and gaps observed in and outside the crane plot, which suggests a high rate of patch turnover, effectively limiting species accumulation. Similar to the edge vs. core comparison, the interpretation of the differences between Brazilian core and Panamanian transects is limited due to low number of study sites. Further studies including forests along environmental and productivity gradients are necessary for a better assessment of the relationship between aboveground biomass turnover of trees and epiphyte communities. Alternatively, studies could assess such relationship by incorporating age as an additional substrate characteristic, as substrates with similar diameter may differ in age and thus time available for colonization. While data on age of tropical trees are scarce, this topic has received increasing attention, with age estimation methods spanning from allometric relationships, over counting rings to isotope dating [51–53]. Meanwhile, studies monitoring epiphytes information could extend their scope to monitor branches (with and without epiphytes). This branch monitoring would provide data on the time of occurrence of key events of substrate dynamics, such as formation, diameter growth and fall of branches. Hence, monitoring branches since their formation, and thus knowing their age, would give the time that these branches had been available for epiphyte colonization. If branches are also monitored on the forest floor, a complete appraisal of branch dynamics could provide further insights into the role of branch dynamics to epiphyte communities.

## Conclusions

Sampling the forest floor for epiphytes constitutes a fast method that can provide, besides floristic data, useful information on epiphyte diversity, community composition and structure, as highlighted by the comparisons with canopy data available for our Panamanian transects. Furthermore, this method requires less work and training effort, is cheaper and safer than climbing techniques and canopy cranes, which can open new avenues for investigations of some aspects of epiphyte demography. This is particularly valuable for those epiphytes occurring in the least accessible, thinnest branches of tree crowns. In this sense, our results confirmed branchfall as a main cause of epiphyte fall. This effect poses demographic constraints on epiphyte populations by increasing mortality (see also [15]) and by reducing time for colonization and for sexual maturation. Consequently, branchfall acts as a strong demographical filter for epiphyte populations. Moreover, branch diameter is a key factor of this demographic filter because branchfall decreases with branch diameter. This is truly independent of local forest dynamics, making demographic filtering greatest in the thin branches of the canopy. In this highly dynamic environment, only small, stress-tolerant and fast growing species are able to recruit, survive and reproduce. Therefore, branchfall plays a key role in structuring the spatial distribution of epiphytic communities.

## Supporting Information

S1 Dataset Tree data. (TXT)

S2 Dataset Branch data. (TXT)

S3 Dataset Epiphyte data on the forest floor. (TXT)

S4 Dataset Epiphyte canopy data. (TXT)

S1 File R code for analyses. (R)

S2 File Epiphytes inventoried within the whole crane plot (c. 0.9 ha) over the branch diameter. (DOC)

S1 Table List of vascular holoepiphytes found on the forest floor per study sites. (DOC)

S2 Table List of vascular holoepiphytes found in the canopy of transects, of the entire Brazilian fragment and of the Panamanian crane site. (DOC)

S3 Table Full transect data and comparison between study sites. (DOC)

S4 Table Generalized additive mixed-effects models (GAMMs) investigating the influence of branch diameter on different variables. (DOC)

S5 Table Indicator species for the compositional difference between ground and canopy at Panamanian transects. (DOC)
