# Supplementary material for: Branchfall as a Demographic Filter for Epiphyte Communities: Lessons from Forest Floor-Based Sampling
Source: PLoS One. 2015 Jun 17;10(6):e0128019. doi: 10.1371/journal.pone.0128019 (PMC4470510; doi:10.1371/journal.pone.0128019)
Supplement: S2 File — (DOC) [file pone.0128019.s006.doc]

**S1 Figure. Epiphytes inventoried within the whole crane plot (c. 0.9 ha) over the branch diameter.** A) Abundance of individuals. B) Species richness. We limited the substrate to 10 cm in diameter for visual comparison with trends found on the forest floor. For raw data, please contact co-author Prof. Gerhard Zotz (University of Oldenburg).
