# Supplementary material for: Branchfall as a Demographic Filter for Epiphyte Communities: Lessons from Forest Floor-Based Sampling
Source: PLoS One. 2015 Jun 17;10(6):e0128019. doi: 10.1371/journal.pone.0128019 (PMC4470510; doi:10.1371/journal.pone.0128019)
Supplement: S1 Table — (DOC) [file pone.0128019.s007.doc]

**S1 Table. List of vascular holoepiphytes found on the forest floor per study site.**

| **Brazil core** | | **Brazil edge** | | **Panama** | |
| --- | --- | --- | --- | --- | --- |
| **Family** | **Species** | **Family** | **Species** | **Family** | **Species** |
| Bromeliaceae | ***Aechmea* cf. *stelligera*** | Bromeliaceae | ***Aechmea* cf. *stelligera*** | Araceae | ***Anthurium acutangulum*** |
|  | ***Tillandsia bulbosa*** |  | ***Tillandsia bulbosa*** |  | ***Anthurium friedrichsthallii*** |
|  | ***Tillandsia juncea*** |  | *Tillandsia juncea* |  | *Anthurium hacumense* |
|  | ***Tillandsia tenuifolia*** |  | ***Tillandsia tenuifolia*** |  | ***Anthurium scandens*** |
|  | ***Tillandsia usneoides*** |  | *Tillandsia usneoides* |  | ***Stenospermation angustifolium*** |
| Cactaceae | *Rhypsalis baccifera* | Orchidaceae | ***Campylocentrum crassyrhyzum*** | Aspleniaceae | ***Asplenium serratum*** |
| Orchidaceae | *Anathallis sclerophylla* |  | ***Cattleya labiata*** | Bromeliaceae | ***Catopsis sessiliflora*** |
|  | ***Campylocentrum crassyrhyzum*** |  | ***Dichaea panamensis*** |  | *Guzmania subcorymbosa* |
|  | ***Cattleya labiata*** |  | ***Dimerandra emarginata*** |  | *Tillandsia anceps* |
|  | ***Dichaea panamensis*** |  | ***Epidendrum difforme*** |  | ***Tillandsia bulbosa*** |
|  | ***Dimerandra emarginata*** |  | ***Epidendrum nocturnum*** | Cactaceae | *Epiphyllum phyllanthus* |
|  | ***Epidendrum difforme*** |  | ***Gomesa barbata*** | Gesneriaceae | ***Codonanthe macradenia*** |
|  | *Epidendrum riggidum* |  | ***Polystachya concreta*** | Orchidaceae | ***Campylocentrum micranthum*** |
|  | ***Gomesa barbata*** |  | ***Rodrighezia bahiensis*** |  | ***Catasetum viridiflavum*** |
|  | *Maxillaria ochroleuca* |  | *Scaphyglottis fusiformis* |  | ***Christensonella uncata*** |
|  | ***Notylia lyrata*** |  | ***Scaphyglottis sickii*** |  | ***Dichaea panamensis*** |
|  | *Polystachya concreta* |  |  |  | ***Epidendrum difforme*** |
|  | ***Prosthechea alagoensis*** |  |  |  | ***Epidendrum nocturnum*** |
|  | ***Prosthechea fragrans*** |  |  |  | *Heterotaxis sessilis* |
|  | ***Rodrighezia bahiensis*** |  |  |  | *Masdevallia livingstoneana* |
|  | ***Scaphyglottis fusiformis*** |  |  |  | ***Mormodes powellii*** |
|  | ***Scaphyglottis sickii*** |  |  |  | ***Polystachya foliosa*** |
|  | *Trigonidium acuminatum* |  |  |  | ***Prosthechea aemula*** |
|  |  |  |  |  | ***Scaphyglottis behrii*** |
|  |  |  |  |  | ***Scaphyglottis longicaulis*** |
|  |  |  |  |  | ***Sobralia fenzliana*** |
|  |  |  |  |  | *Sobralia fragans* |
|  |  |  |  |  | ***Trichocentrum capistratum*** |
|  |  |  |  |  | *Trichopilia maculata* |
|  |  |  |  |  | *Trichosalpinx orbicularis* |
|  |  |  |  |  | ***Trigonidium egertonianum*** |
|  |  |  |  | Piperaceae | *Peperomia cordulata* |
|  |  |  |  |  | ***Peperomia rotundifolia*** |
|  |  |  |  | Polypodiaceae | ***Dicranoglossum panamense*** |
|  |  |  |  |  | ***Microgramma lycopodioides*** |
|  |  |  |  |  | ***Microgramma percussa*** |
|  |  |  |  |  | ***Niphidium crassifolium*** |
|  |  |  |  | Vittariaceae | ***Ananthacorus angustifolius*** |
|  |  |  |  |  | ***Vittaria lineata*** |
| Total | 23 |  | 16 |  | 27 |

We did not surveyed ferns and aroids at the Brazilian sites. Species names follow the The Plant List (http://www.theplantlist.org/). Species found attached to branches are in bold. Vouchers of Brazilian species were deposited in the herbarium of the Federal University of Pernambuco and in the herbarium of the Federal University of Paraiba (Areia campus, [1]). Vouchers of the Panamanian species were deposited in the herbarium of the Smithsonian Tropical Research Institute, Panama (Tupper Center, [2]).

**References**

1. Siqueira Filho JA, Felix LP. Bromélias e orquídeas. In: Porto KC, de Almelda-Cortez JS, Tabarelli M, editors. Diversidade Biológica do Centro de endemismo Pernambuco. Brasília: Ministério do Meio Ambiente; 2005. pp. 123-133.

2. Zotz G, Schultz S. The vascular epiphytes of a lowland forest in Panama-species composition and spatial structure. Plant Ecol 2008;195: 131-141.
