# Supplementary material for: Branchfall as a Demographic Filter for Epiphyte Communities: Lessons from Forest Floor-Based Sampling
Source: PLoS One. 2015 Jun 17;10(6):e0128019. doi: 10.1371/journal.pone.0128019 (PMC4470510; doi:10.1371/journal.pone.0128019)
Supplement: S2 Table — (DOC) [file pone.0128019.s008.doc]

**S2 Table. List of vascular holoepiphytes found in the canopy per forest.**

| **Brazil** | | **Panama** | |
| --- | --- | --- | --- |
| **Family** | **Species** | **Family** | **Species** |
| Bromeliaceae | ***Aechmea stelligera**** | Araceae | ***Anthurium acutangulum***^†^ |
|  | ***Aechmea fulgens*** |  | ***Anthurium bakeri*** |
|  | ***Bilbergia morelii*** |  | ***Anthurium brownii***^†^ |
|  | *Canistrum alagoanum* |  | ***Anthurium clavigerum***^†^ |
|  | *Guzmania lingulata* |  | ***Anthurium durandii***^†^ |
|  | *Lymania smithii* |  | ***Anthurium friedrichsthalii***^†^ |
|  | ***Tillandsia bulbosa*** |  | ***Anthurium hacumense***^†^ |
|  | ***Tillandsia juncea**** |  | ***Anthurium scandens***^†^ |
|  | *Tillandsia stricta* |  | ***Philodendron radiatum***^†^ |
|  | ***Tillandsia tenuifolia**** |  | ***Philodendron sagittifolium***^†^ |
|  | ***Tillandsia usneoides**** |  | ***Stenospermation angustifolium*** |
| Cactaceae | ***Rhypsalis baccifera*** | Aspleniaceae | ***Asplenium juglandifolium*** |
|  | ***Epiphyllum phyllanthus*** |  | ***Asplenium serratum***^†^ |
| Orchidaceae | ***Acianthera pernambucensis*** | Bromeliaceae | ***Aechmea tillandsioides***^†^ |
|  | *Anathallis brevipes* |  | ***Catopsis sessiliflora***^†^ |
|  | ***Anathallis sclerophylla*** |  | *Guzmania musaica* |
|  | *Campylocentrum amazonicum* |  | ***Guzmania subcorymbosa***^†^ |
|  | ***Campylocentrum crassyrhyzum**** |  | ***Tillandsia anceps***^†^ |
|  | *Catasetum macrocarpum* |  | ***Tillandsia bulbosa***^†^ |
|  | *Cattleya granulosa* |  | ***Vriesea gladioliflora***^†^ |
|  | ***Cattleya labiata**** |  | *Vriesea sanguinolenta* |
|  | ***Dichaea panamensis**** | Cactaceae | ***Epiphyllum phyllanthus***^†^ |
|  | ***Dimerandra emarginata**** |  | ***Hylocereus monacanthus***^†^ |
|  | *Encyclia longifolia* |  | ***Weberocereus tunilla***^†^ |
|  | ***Epidendrum difforme**** | Gesneriaceae | ***Codonanthe macradenia***^†^ |
|  | ***Epidendrum nocturnum**** |  | *Columnea billbergiana* |
|  | *Epidendrum ramosum** |  | *Drymonia serrulata* |
|  | ***Epidendrum riggidum*** | Hymenophyllaceae | ***Hymenophyllum brevifrons*** |
|  | *Heterotaxis discolor* |  | ***Trichomanes anadromum***^†^ |
|  | *Jacquiniella globosa* |  | ***Trichomanes angustifrons***^†^ |
|  | ***Maxillaria ochroleuca*** |  | ***Trichomanes godmanii***^†^ |
|  | ***Notylia lyrata*** |  | ***Trichomanes nummularium***^†^ |
|  | ***Gomesa barbata**** |  | ***Trichomanes ovale***^†^ |
|  | ***Polystachya concreta**** |  | ***Trichomanes punctatum***^†^ |
|  | ***Prosthechea alagoensis*** | Lomariopsidaceae | ***Elaphoglossum herminieri*** |
|  | ***Prosthechea fragrans*** |  | ***Elaphoglossum sporadolepis***^†^ |
|  | ***Rodrighezia bahiensis**** | Orchidaceae | ***Acianthera verecunda***^†^ |
|  | *Scaphyglottis emarginata* |  | ***Aspasia principissa*** |
|  | ***Scaphyglottis fusiformis**** |  | *Camaridium sp.* |
|  | ***Scaphyglottis sickii**** |  | ***Campylocentrum micranthum***^†^ |
|  | *Stellis clorantha* |  | ***Catasetum viridiflavum***^†^ |
|  | ***Stellis filiformis*** |  | ***Caularthron bilamellatum***^†^ |
|  | *Trichocentrum fuscum** |  | ***Christensonella uncata***^†^ |
|  | ***Trigonidium acuminatum*** |  | *Cochleanthes lipscombiae* |
| Piperaceae | *Peperomia aff. circinata* |  | *Cryptarrhena guatemalensis* |
|  | *Peperomia macrostachya* |  | ***Dichaea panamensis***^†^ |
|  | ***Peperomia pellucida*** |  | ***Dimerandra emarginata***^†^ |
|  | *Peperomia sp.* |  | ***Elleanthus longibracteatus*** |
|  |  |  | *Epidendrum coronatum* |
|  |  |  | ***Epidendrum difforme***^†^ |
|  |  |  | *Epidendrum imatophyllum* |
|  |  |  | ***Epidendrum nocturnum***^†^ |
|  |  |  | *Epidendrum rousseauae* |
|  |  |  | ***Epidendrum schlechterianum***^†^ |
|  |  |  | ***Gongora quinquenervis***^†^ |
|  |  |  | ***Heterotaxis discolor*** |
|  |  |  | *Heterotaxis sessilis* |
|  |  |  | ***Jacquiniella pedunculata***^†^ |
|  |  |  | *Jacquiniella sp.* |
|  |  |  | *Kefersteinia sp.* |
|  |  |  | ***Lockhartia acuta***^†^ |
|  |  |  | *Lockhartia pittieri* |
|  |  |  | *Macradenia brassavolae* |
|  |  |  | ***Masdevallia livingstoneana*** |
|  |  |  | *Maxillariella acervata* |
|  |  |  | *Mormodes powellii* |
|  |  |  | ***Notylia albida***^†^ |
|  |  |  | *Oncidium lineoligerum* |
|  |  |  | *Ornithocephalus sp.* |
|  |  |  | ***Polystachya foliosa***^†^ |
|  |  |  | ***Specklinia brighamii***^†^ |
|  |  |  | ***Specklinia grobyi***^†^ |
|  |  |  | *Prosthechea aemula* |
|  |  |  | *Prosthechea chacaoensis* |
|  |  |  | *Prosthechea chimborazoensis* |
|  |  |  | ***Rossioglossum ampliatum***^†^ |
|  |  |  | ***Scaphyglottis behrii***^†^ |
|  |  |  | ***Scaphyglottis longicaulis***^†^ |
|  |  |  | ***Scaphyglottis prolifera***^†^ |
|  |  |  | ***Sobralia fenzliana*** |
|  |  |  | ***Sobralia fragans***^†^ |
|  |  |  | ***Stelis crescentiicola***^†^ |
|  |  |  | ***Trichocentrum capistratum***^†^ |
|  |  |  | ***Trichopilia maculata***^†^ |
|  |  |  | ***Trichosalpinx orbicularis*** |
|  |  |  | ***Trigonidium egertonianum*** |
|  |  | Piperaceae | ***Peperomia cordulata***^†^ |
|  |  |  | ***Peperomia ebingeri*** |
|  |  |  | *Peperomia macrostachya* |
|  |  |  | ***Peperomia obtusifolia***^†^ |
|  |  |  | ***Peperomia rotundifolia***^†^ |
|  |  | Polypodiaceae | ***Campyloneurum aphanophlebium***^†^ |
|  |  |  | ***Campyloneurum phylitidis***^†^ |
|  |  |  | ***Dicranoglossum panamense***^†^ |
|  |  |  | ***Microgramma lycopodioides***^†^ |
|  |  |  | ***Microgramma percussa***^†^ |
|  |  |  | *Microgramma reptans* |
|  |  |  | ***Niphidium crassifolium***^†^ |
|  |  |  | *Pecluma pectinata* |
|  |  |  | *Serpocaulon triseriale* |
|  |  |  | ***Serpocaulon wagneri*** |
|  |  | Selaginellaceae | *Huperzia dichotoma* |
|  |  | Vittariaceae | ***Ananthacorus angustifolius***^†^ |
|  |  |  | ***Anetium citrifolium***^†^ |
|  |  |  | ***Antrophyum lanceolatum***^†^ |
|  |  |  | ***Hecistopteris pumila*** |
|  |  |  | ***Vittaria lineata*** |

* Species found in the Brazilian edge transects are indicated with asterisks.

^†^ For Panamanian transect canopies, species occurring on substrate <10 cm in diameter.

Species names follow the The Plant List (http://www.theplantlist.org/). The list of both Brazilian study sites is the same (ferns and aroids were not surveyed), based on [1], whereas the list of the Panamanian study site is based on previous inventories [Glenda Mendieta-Leiva & Gerhard Zotz, unpublished data; 2]. Species found in the canopy above Brazilian core and Panamanian transects are indicated in bold. Transect canopy information was based on own observations in the Brazilian sites (see main text) and on inventoried data in the Panamanian site (Glenda Mendieta-Leiva & Gerhard Zotz, unpublished data). Vouchers of Brazilian species were deposited in the herbarium of the Federal University of Pernambuco and in the herbarium of the Federal University of Paraiba (Areia campus, [1]). Vouchers of the Panamanian species were deposited in the herbarium of the Smithsonian Tropical Research Institute, Panama (Tupper Center, [2]).

**References**

1. Siqueira Filho JA, Felix LP. Bromélias e orquídeas. In: Porto KC, de Almelda-Cortez JS, Tabarelli M, editors. Diversidade Biológica do Centro de endemismo Pernambuco. Brasília: Ministério do Meio Ambiente; 2005. pp. 123-133.

2. Zotz G, Schultz S. The vascular epiphytes of a lowland forest in Panama-species composition and spatial structure. Plant Ecol 2008;195: 131-141.
