# Supplementary material for: Branchfall as a Demographic Filter for Epiphyte Communities: Lessons from Forest Floor-Based Sampling
Source: PLoS One. 2015 Jun 17;10(6):e0128019. doi: 10.1371/journal.pone.0128019 (PMC4470510; doi:10.1371/journal.pone.0128019)
Supplement: S3 Table — (DOC) [file pone.0128019.s009.doc]

**S3 Table. Full transect data and comparisons between study sites.**

| **Variable** | | **Brazilian core transects (*n*=30)** | **Brazilian edge transects (*n*=30)** | **Panamanian transects (*n*=36)** | | |
| --- | --- | --- | --- | --- | --- | --- |
|  | |  |  | **No ferns and aroids** | **All species** | |
| **Trees** | **Total** | 184 | 154 | 171 | | |
|  | **Mean number per transect** | 5.1 ± 2.1 ab | 6.4 ± 3.2 a | 4.8 ± 2.1 b | | |
|  | **Mean DBH (m)** | 0.2 ± 0.1 a | 0.18 ± 0.06 b | 0.16 ± 0.07 b† | | |
|  | **Mean height at first branching (m)** | 8.2 ± 2.0 a | 4.7 ± 2.0 b | 7.8 ± 2.4 a† | | |
|  | **Mean height (m)** | 15.4 ± 3.4 a | 10.9 ± 2.7 b | 12.7 ± 3.3 c† | | |
| **Branch abundance** | **Total** | 9759 | 6721 | 7939 | | |
|  | **Mean per transect** | 325 ± 284 | 224 ± 102 | 220 ± 169 | | |
|  | **Mean per m^2^** | 130 ± 114 | 90 ± 41 | 88 ± 68 | | |
| **Epiphyte abundance** | **Total** | 546 | 349 | 164 | | 232 |
|  | **Mean per transect** | 18.2 ± 20.5 a | 11.6 ± 17.8 ab | 4.6 ± 7.1 b | | 6.4 ± 9.8 |
|  | **Mean per m^2^** | 0.36 ± 0.41 a | 0.23 ± 0.36 ab | 0.11 ± 0.15 b | | 0.13 ± 0.20 |
|  | **Total adults** | 211 | 153 | 86 | | 101 |
|  | **Mean adults per transect** | 7.0 ± 8.8 a | 5.1 ± 11.8 ab | 2.4 ± 4.1 b | | 2.8 ± 4.7 |
|  | **Mean adults per m^2^** | 0.14 ± 0.18 a | 0.10 ± 0.24 ab | 0.05 ± 0.08 b | | 0.06 ± 0.09 |
| **Epiphyte abundance (detached from branches)** | **Total** | 179 | 89 | 52 | | 68 |
|  | **Mean per transect** | 6.0 ± 4.9 a | 3.0 ± 7.2 ab | 1.4 ± 4.3 b | | 1.9 ± 6.0 |
|  | **Mean per m^2^** | 0.12 ± 0.10 a | 0.06 ± 0.14 ab | 0.03 ± 0.09 b | | 0.04 ± 0.12 |
|  | **Total adults** | 100 | 57 | 35 | | 42 |
|  | **Mean adults per transect** | 3.3 ± 3.2 a | 1.9 ± 6.2 ab | 1.0 ± 3.1 b | | 1.2 ± 3.8 |
|  | **Mean adults per m^2^** | 0.07 ± 0.06 a | 0.04 ± 0.12 ab | 0.02 ± 0.06 b | | 0.02 ± 0.08 |
| **Epiphyte abundance (attached to branches)** | **Total** | 367 | 260 | 112 | | 164 |
|  | **Mean per transect** | 12.2 ± 17.0 a | 8.7 ± 16.8 ab | 3.1 ± 5.1 b | | 4.6 ± 6.4 |
|  | **Mean per m^2^** | 0.24 ± 0.34 a | 0.17 ± 0.34 ab | 0.06 ± 0.10 b | | 0.09 ± 0.13 |
|  | **Mean per transect per branch** | 2.8 10^-3^ ± 3.5 10^-3^ | 2.2 10^-3^ ± 4.5 10^-3^ | 1.1 10^-3^ ± 2.0 10^-3^ | | 1.8 10^-3^ ± 3.1 10^-3^ |
|  | **Total adults** | 1111 | 96 | 51 | | 59 |
|  | **Mean adults per transect** | 3.7 ± 6.3 | 3.20± 10.3 | 1.4 ± 2.5 | | 1.6 ± 2.5 |
|  | **Mean adults per m^2^** | 0.07 ± 0.13 | 0.06 ± 0.20 | 0.03 ± 0.05 | | 0.03 ± 0.05 |
|  | **Mean adults per transect per branch** | 8.5 10^-4^ ± 1.5 10^-3^ | 8.8 10^-4^ ± 2.4 10^-3^ | 6.7 10^-4^ ± 1.3 10^-3^ | | 7.5 10^-4^ ± 1.3 10^-3^ |
| **Epiphyte richness (total)** | **Total** | 23 | 16 | 27 | | 39 |
|  | **Mean per transect** | 5.1 ± 3.2 a | 2.3 ± 1.9 b | 1.9 ± 2.3 b | | 2.8 ± 3.6 |
|  | **Mean per m^2^** | 0.10 ± 0.06 a | 0.05 ± 0.04 b | 0.04 ± 0.05 b | | 0.06 ± 0.07 |
|  | **Total adults** | 21 | 14 | 17 | | 24 |
|  | **Mean adults per transect** | 3.3 ± 2.7 a | 1.1 ± 1.3 b | 1.1 ± 1.6 b | | 1.4 ± 2.0 |
|  | **Mean adults per m^2^** | 0.07 ± 0.05 a | 0.02 ± 0.02 b | 0.02 ± 0.03 b | | 0.03 ± 0.04 |
| **Epiphyte richness (detached from branches)** | **Total** | 20 | 10 | 16 | | 23 |
|  | **Mean per transect** | 3.0 ± 2.4 a | 0.7 ± 1.1 b | 0.6 ± 1.4 b | | 0.9 ± 2.4 |
|  | **Mean per m^2^** | 0.06 ± 0.05 a | 0.015 ± 0.02 b | 0.01 ± 0.03b | | 0.02 ± 0.05 |
|  | **Total adults** | 18 | 9 | 10 | | 13 |
|  | **Mean adults per transect** | 2.3 ± 1.9 a | 0.4 ± 0.7 b | 0.4 ± 1.0 b | | 0.6 ± 1.5 |
|  | **Mean adults per m^2^** | 0.05 ± 0.04 a | 0.007 ± 0.014 b | 0.008 ± 0.02 b | | 0.01 ± 0.03 |
| **Epiphyte richness (attached to branches)** | **Total** | 17 | 13 | 18 | | 29 |
|  | **Mean per transect** | 3.3 ± 2.5 a | 1.8 ± 1.8 b | 1.4 ± 1.6 b | | 2.2 ± 2.3 |
|  | **Mean per m^2^** | 0.06 ± 0.05 a | 0.38 ± 0.35 b | 0.03 ± 0.03 b | | 0.04 ± 0.05 |
|  | **Mean per transect per branch** | 7.7 10^-4^ ± 7.1 10^-4^ | 5.2 10^-4^ ± 5.5 10^-4^ | 5.3 10^-4^ ± 7.5 10^-4^ | | 7.3 10^-4^ ± 7.9 10^-4^ |
|  | **Total adults** | 16 | 11 | 12 | | 18 |
|  | **Mean adults per transect** | 1.57 ± 2.0 | 0.80 ± 1.14 | 0.72 ± 1.08 | | 0.94 ± 1.24 |
|  | **Mean adults per m^2^** | 0.03 ± 0.04 | 0.015 ± 0.02 | 0.014 ± 0.02 | | 0.019 ± 0.02 |
|  | **Mean adults per transect per branch** | 3.3 10^-4^ ± 4.6 10^-4^ | 1.9 10^-4^ ± 3.0 10^-4^ | 2.8 10^-4^ ± 4.5 10^-4^ | | 3.2 10^-4^ ± 4.2 10^-4^ |

† indicates n=35 (excluding one transect without trees)

Several measures characterizing forest structure, branch abundance, as well as epiphyte abundance and richness were standardized per transect and their means were compared with simultaneous max-*t* tests using Tukey contrasts that are robust under non-normality, heteroscedasticity and variable sample size [1]. The standardized measures ± SD are given below. Significantly different means are followed by different letters representing pairwise differences. Additionally, total numbers per study site are provided. We show values with both the transect and m^2^ as unit area. Note that for epiphytes, only mean values for Panamanian transects without ferns and aroids were used in the comparisons with Brazilian study sites.

**References**

1. Herberich E, Sikorski J, Hothorn T. A robust procedure for comparing multiple means under heteroscedasticity in unbalanced designs. Plos One 2010;5: e9788.
