# Supplementary material for: Branchfall as a Demographic Filter for Epiphyte Communities: Lessons from Forest Floor-Based Sampling
Source: PLoS One. 2015 Jun 17;10(6):e0128019. doi: 10.1371/journal.pone.0128019 (PMC4470510; doi:10.1371/journal.pone.0128019)
Supplement: S4 Table — (DOC) [file pone.0128019.s010.doc]

**S4 Table. Generalized additive mixed-effects models (GAMMs) investigating the influence of branch diameter on different variables.**

| **Study site** | **Effect** | | **Branch abundance** | **Abs. epiphyte abundance** | **Abs. epiphyte richness** | **Epiphyte abundance/branch** | **Epiphyte richness/branch** | **Proportion of adults** |
| --- | --- | --- | --- | --- | --- | --- | --- | --- |
| **Brazil core** | **Diameter class** | **df_eff_** | 1.00 | 1.98 | 1.96 | 1.00 | 1.00 | 1.00 |
|  |  | **Trend** | negative*** | unimodal*** | unimodal*** | positive*** | positive*** | positive *** |
|  | **Transect** | **df_eff_** | 0 | 25*** | 23*** | 9* | 8 | 10*** |
| **Brazil edge** | **Diameter class** | **df_eff_** | 1.89 | 1.00 | 1.94 | 1.00 | 1.00 | 1.00 |
|  |  | **Trend** | negative*** | none | unimodal*** | positive*** | positive*** | positive * |
|  | **Transect** | **df_eff_** | 0 | 26*** | 19*** | 5 | 6* | 13*** |
| **Panama** | **Diameter class** | **df_eff_** | 1.00 | 1.87 | 1.91 | 1.54 | 1.64 | 1.60 |
|  |  | **Trend** | negative*** | positive*** | positive*** | positive*** | positive*** | positive *** |
|  | **Transect** | **df_eff_** | 0.84* | 28*** | 22*** | 8* | 8* | 4* |

* 0.05 >*P*>0.01;** 0.01 >*P*>0.001; *** *P*< 0.0001

The table provides the effective degrees of freedom (df_eff_) for both fixed (diameter class) and random (transect) effects as well as the trend of the relationship over branch diameter. The df_eff_ reflect the ruggedness of the smoothing parameter [1]. For absolute branch abundance (branches per transect), epiphyte abundance (individuals per transect), richness (species per transect) and proportion of adults, all transects were considered (*n*=30, 30 and 36 for Brazilian core, Brazilian edge and Panamanian transects, respectively), whereas for epiphyte abundance and richness per branch, only transects with epiphytes were considered (*n*= 26, 21 and 25 for Brazilian core, Brazilian edge and Panamanian transects, respectively). Significant *P*-values (α= 5%) are indicated with asterisks.

**References**

1. Zuur A, Ieno EN, Walker N, Saveliev AA, Smith GM. Mixed effects models and extensions in ecology with R. New York, NY, USA: Springer, 2009.
