# Supplementary material for: Branchfall as a Demographic Filter for Epiphyte Communities: Lessons from Forest Floor-Based Sampling
Source: PLoS One. 2015 Jun 17;10(6):e0128019. doi: 10.1371/journal.pone.0128019 (PMC4470510; doi:10.1371/journal.pone.0128019)
Supplement: S5 Table — (DOC) [file pone.0128019.s011.doc]

**S5 Table. Indicator species for the compositional difference between ground and canopy at Panamanian transects.**

| **Family** | **Species** |
| --- | --- |
| Araceae | ***Anthurium acutangulum*** |
|  | ***Anthurium clavigerum*** |
|  | *Anthurium friedrichsthalii* |
|  | *Anthurium hacumense* |
| Bromeliaceae | ***Vriesea gladioliflora*** |
| Hymenophyllaceae | *Trichomanes angustifrons* |
|  | *Trichomanes nummularium* |
|  | ***Trichomanes ovale*** |
|  | *Trichomanes punctatum* |
| Lomariopsidaceae | *Elaphoglossum sporadolepis* |
| Orchidaceae | *Dichaea panamensis* |
|  | ***Polystachya foliosa*** |
|  | *Scaphyglottis longicaulis* |
| Polypodiaceae | ***Campyloneurum aphanophlebium*** |
|  | *Campyloneurum phylitidis* |
|  | ***Dicranoglossum panamense*** |
|  | ***Niphidium crassifolium*** |
| Vittariaceae | *Ananthacorus angustifolius* |
|  | ***Anetium citrifolium*** |

We performed a Dufrene-Legendre indicator species analysis to assess the species that contributed to the significant difference in species composition between the epiphytes found on the forest floor and in the canopy. All listed species were indicatory of canopy composition in the analysis considering all epiphytes (including epiphytes found on the forest floor detached from branches and in the entire canopy). Indicator species resulting from the analysis considering only epiphytes on substrate < 10 cm in diameter are indicated in bold.
